# Evaluating Drug Prices, Availability, Affordability, and Price Components: Implications for Access to Drugs in Malaysia

**DOI:** 10.1371/journal.pmed.0040082

**Published:** 2007-03-27

**Authors:** Zaheer Ud Din Babar, Mohamed Izham Mohamed Ibrahim, Harpal Singh, Nadeem Irfan Bukahri, Andrew Creese

**Affiliations:** 1 Discipline of Social and Administrative Pharmacy, School of Pharmaceutical Sciences, Universiti Sains Malaysia, Penang, Malaysia; 2 School of Pharmaceutical Sciences, University College Sedaya International, Kuala Lumpur, Malaysia; 3 School of Pharmacy, International Medical University, Kuala Lumpur, Malaysia; 4 La Grille, Les Ventes de Bourse, France; La Trobe University Melbourne, Australia

## Abstract

**Background:**

Malaysia's stable health care system is facing challenges with increasing medicine costs. To investigate these issues a survey was carried out to evaluate medicine prices, availability, affordability, and the structure of price components.

**Methods and Findings:**

The methodology developed by the World Health Organization (WHO) and Health Action International (HAI) was used. Price and availability data for 48 medicines was collected from 20 public sector facilities, 32 private sector retail pharmacies and 20 dispensing doctors in four geographical regions of West Malaysia. Medicine prices were compared with international reference prices (IRPs) to obtain a median price ratio. The daily wage of the lowest paid unskilled government worker was used to gauge the affordability of medicines. Price component data were collected throughout the supply chain, and markups, taxes, and other distribution costs were identified. In private pharmacies, innovator brand (IB) prices were 16 times higher than the IRPs, while generics were 6.6 times higher. In dispensing doctor clinics, the figures were 15 times higher for innovator brands and 7.5 for generics. Dispensing doctors applied high markups of 50%–76% for IBs, and up to 316% for generics. Retail pharmacy markups were also high—25%–38% and 100%–140% for IBs and generics, respectively. In the public sector, where medicines are free, availability was low even for medicines on the National Essential Drugs List. For a month's treatment for peptic ulcer disease and hypertension people have to pay about a week's wages in the private sector.

**Conclusions:**

The free market by definition does not control medicine prices, necessitating price monitoring and control mechanisms. Markups for generic products are greater than for IBs. Reducing the base price without controlling markups may increase profits for retailers and dispensing doctors without reducing the price paid by end users. To increase access and affordability, promotion of generic medicines and improved availability of medicines in the public sector are required.

## Abstract 2

Drug price and availability data were collected from West Malaysian public sector facilities, private sector retail pharmacies, and dispensing doctors. Mark-ups were higher on generic drugs than on innovator brands.

## Editors' Summary

**Background.:**

The World Health Organization has said that one-third of the people of the world cannot access the medicines they need. An important reason for this problem is that prices are often too high for people or government-funded health systems to afford. In developing countries, most people who need medicines have to pay for them out of their own pockets. Where the cost of drugs is covered by health systems, spending on medicines is a major part of the total healthcare budget. Governments use a variety of approaches to try to control the cost of drugs and make sure that essential medicines are affordable and not overpriced. According to the theory of “free market economics,” the costs of goods and services are determined by interactions between buyers and sellers and not by government intervention. However, free market economics does not work well at containing the costs of medicines, particularly new medicines, because new medicines are protected by patent law, which legally prevents others from making, using, or selling the medicine for a particular period of time. Therefore, without government intervention, there is nothing to help to push down prices.

**Why Was This Study Done?:**

Malaysia is a middle-income country with a relatively effective public health system, but it is facing a rapid rise in drug costs. In Malaysia, medicine prices are determined by free-market economics, without any control by government. Government hospitals are expected to provide drugs free, but a substantial proportion of medicines are paid for by patients who buy them directly from private pharmacies or prescribing doctors. There is evidence that Malaysian patients have difficulties accessing the drugs they need and that cost is an important factor. Therefore, the researchers who wrote this paper wanted to examine the cost of different medicines in Malaysia, and their availability and affordability from different sources.

**What Did the Researchers Do and Find?:**

In this research project, 48 drugs were studied, of which 28 were part of a “core list” identified by the World Health Organization as “essential drugs” on the basis of the global burden of disease. The remaining 20 reflected health care needs in Malaysia itself. The costs of each medicine were collected from government hospitals, private pharmacies, and dispensing doctors in four different regions of Malaysia. Data were collected for the “innovator brand” (made by the original patent holder) and for “generic” brands (an equivalent drug to the innovator brand, produced by a different company once the innovator brand no longer has an exclusive patent). The medicine prices were compared against international reference prices (IRP), which are the average prices offered by not-for-profit drug companies to developing countries. Finally, the researchers also compared the cost of the drugs with daily wages, in order to work out their “affordability.”

The researchers found that, irrespective of the source of medicines, prices were on average very much higher than the international reference price, ranging from 2.4 times the IRP for innovator brands accessed through public hospitals, to 16 times the IRP for innovator brands accessed through private pharmacies. The availability of medicines was also very poor, with only 25% of generic medicines available on average through the public sector. The affordability of many of the medicines studied was again very poor. For example, one month's supply of ranitidine (a drug for stomach ulcers) was equivalent to around three days' wages for a low-paid government worker, and one month's supply of fluoxetine (an antidepressant) would cost around 26 days' wages.

**What Do These Findings Mean?:**

These results show that essential drugs are very expensive in Malaysia and are not universally available. Many people would not be able to pay for essential medicines. The cost of medicines in Malaysia seems to be much higher than in areas of India and Sri Lanka, although the researchers did not attempt to collect data in order to carry out an international comparison. It is possible that the high cost and low availability in Malaysia are the result of a lack of government regulation. Overall, the findings suggest that the government should set up mechanisms to prevent drug manufacturers from increasing prices too much and thus ensure greater access to essential medicines.

**Additional Information.:**

Please access these Web sites via the online version of this summary at http://dx.doi.org/10.1371/journal.pmed.0040082.

- Read a related *PLoS Medicine* Perspective article by Suzanne Hill
- Information is available from the World Health Organization on Improving Access to Medicines
- Information on medicine prices is available from Health Action International
- Wikipedia has an entry on Patent (a type of intellectual property that is normally used to prevent other companies from selling a newly invented medicine). (Wikipedia is an internet encyclopedia anyone can edit.)
- The Drugs for Neglected Diseases Initiative is an international collaboration between public organizations that aims to develop drugs for people suffering from neglected diseases

## Introduction

The price of medicine is considered one of the most important obstacles to access [1]. The purchase of medicines contributes significantly to the health care budget of developing countries, and drug expenditures may amount to 50%–90% of nonpersonnel costs [2]. In developing countries, studies and data on medicine prices are scanty. Measuring and understanding the reasons for the price of medicines is the first stage in developing medicine pricing policies that would ensure the affordability of medicines.

In its World Health Report 2000, WHO has ranked Malaysia at 31 among 191 countries for the performance in overall health care and was recommended as a model to other developing countries. One indicator, the “Health Adjusted Life Expectancy” at birth, is comparable to that of industrialized countries [3]. Malaysia has achieved this level by using only a few percentage of its gross domestic product on healthcare [4]. Government spending on health care was RM (Malaysian ringgits) 1 billion (USD 1 = RM 3.82) or 3.6% of the national budget in 1983 and 3.8% in 2001 [5,6]. Russia, even after spending 25% per person more than Malaysia on health care, has reportedly not performed well as indicted by low rankings in a number of indicators [7].

However, this stable health care system is now facing challenges, and increasing health care expenditures on medicines is one of them. In 1995 the government spent more than RM 200 million to procure drugs in public hospitals, which has now increased to RM 800 million annually, but still, faces challenges of access [8,9]. High drug prices could be one of the reasons for this budgetary burden, as it has been an issue and public interest nongovernmental organizations have been lobbying for decades to reduce high medicine prices [10,11].

Malaysia practices a “free market economy” and a “price deregulation system” in which manufacturers, distributors, and retailers set medicine prices without government control. In Malaysia, medicine prices have been reported to escalate even faster than prices in the developed world, and are higher than international prices, indicating high medical costs [12–14]. Although the government provides free medicine in public hospitals, in recent years there are reports that patients are increasingly asked to buy their own medicines [4,15]. Nonavailability of medicines and long waiting hours are other reasons that patients obtain their medicines from private pharmacies and dispensing doctors. A consumer survey showed that 37% of patients obtain medicines from private hospitals or clinics and 42% from retail pharmacies, requiring significant out-of-pocket expenditures [16].

With the annual increase in drug costs and high medicine prices, the government is finding it increasingly hard to finance pharmaceuticals in the public sector. To deal with this budgetary burden, the government is planning to change the current system of free drugs into a pay-per-fee system, whereby private dispensaries in government hospitals will be set up (initially in two public hospitals) to reduce the annual subsidy cost. It is expected that the public may have to pay for medicines obtained from government hospitals in future [9]. If patients are asked to purchase their medicine themselves, the problem of affordability may worsen. In that scenario, the out-of-pocket expenditures will rise further and will pose serious challenges to public health.

To investigate these issues, a study to measure medicine prices, price structure, availability, and affordability was carried out in different sectors—in the public and the private retail pharmacy sectors and in dispensing doctors' clinics. Prices were compared with IRPs.

## Methods

The study followed the WHO–Health Action International (HAI) methodology [1]. Among a total of 48 medicines included in the survey, 28 belong to the core list medicines suggested by WHO–HAI for international comparison, and 20 were added as supplementary drugs. The core list medicines were selected on the basis of the global disease burden. The supplementary list was prepared on the basis of the local disease burden, local needs as determined by a community survey, while other factors, such as drug availability and utilization patterns, were taken into account [17].

Drugs selected for the supplementary list had to have an IRP. The supplementary list was finalized after expert opinion and feedback from international experts (from WHO and HAI) and a national advisory group (a group of practicing pharmacists and doctors, an economist, academicians, officials from Ministry of Health, and a consumer representative. All the medicines included in the study are listed in Table S1.

For each medicine, data were collected on the price and availability of: innovator brand (IB), most-sold generic equivalent (MSG), and lowest-price generic equivalent (LPG). The MSG was estimated nationally through preliminary surveys, while the LPG was determined at the facility level.

A systematic sampling method was used to collect data. Four geographical regions in West Malaysia were selected including the Federal territory of Kuala Lumpur, Penang (northwest), Johore Bahru (southeast) and Kota Bharu (northeast). Areas 2–4 are within 400 kilometres (one day travelling) from Kuala Lumpur (Area 1). These regions are fairly representative of the whole country. In each area, one major city and four peripheral cities were chosen. In each area, the main government hospital in the major city and four other government hospitals in peripheral cities were included. The peripheral area was no more than a two-hour drive from the major city selected. One dispensing doctor and one or two private retail pharmacies were chosen within a five-kilometre radius of the index government hospital. The distribution and the number of all facilities sampled are listed in Table 1. An online map of Malaysia containing the areas mentioned in Table 1 is available on Wikimapia (http://wikimapia.org/#y=3255693&x=101700439&z=8&l=0&m=a&v=2). Data collection was undertaken by pharmacy students, the associate survey manager, and the principal investigator.

**Table 1:**
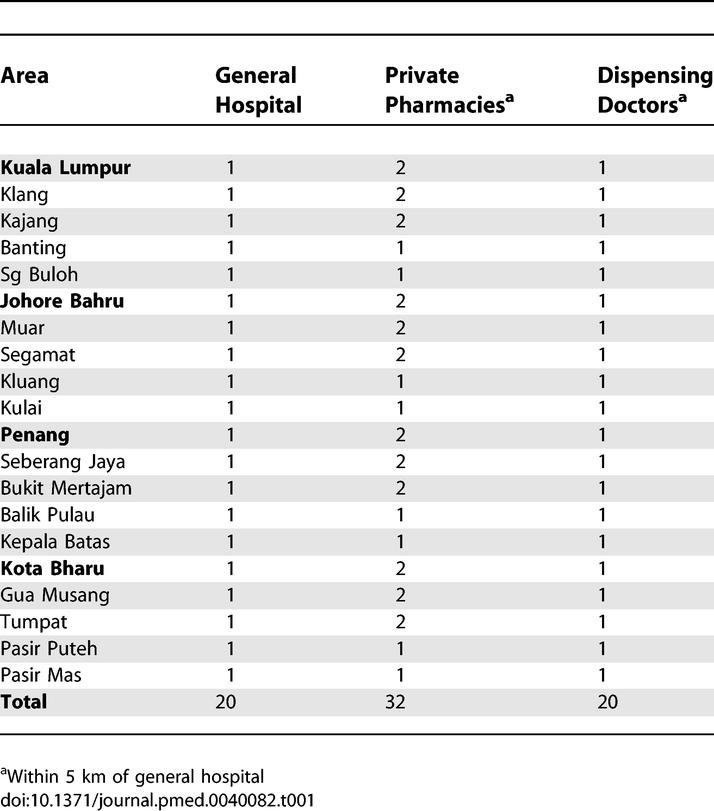
Distribution and Number of Facilities for Sampling Plan

For the purpose of statistical analysis, the drugs found common in all four regions were included. “Median unit price” for each medicine was calculated and then the “median-median” among the four regions for matched sets of medicines was compared. A Kruskal-Wallis test was applied, and *p* < 0.05 was used to indicate a significant difference.

Medicine prices and availability were assessed in public sector facilities, community retail pharmacies, and dispensing doctors' clinics and recorded on the data collection form. The unit prices were calculated, and each entry on the form was checked carefully on the day of data collection. The data collected on the medicine price data collection form were entered by designated personnel into the software International Medicines Price Workbook (v. 3.06). The prices were double entered to ensure accuracy. The Workbook's auto checker was also used to assist in the verification process.

The Workbook software calculated the median price ratio (MPR) for each medicine type in each sector only if the medicine was available in at least four facilities. The MPR was the comparison of the local median unit price of the medicine with the median unit price in the Management Sciences for Health 2003 Price Indicator Guide (the IRP) [18]. The IRPs are the medians of recent procurement or tender prices offered by predominantly not-for-profit suppliers to developing countries for multi-source products [18]. Normally, an MPR of 1 or less is taken as efficient procurement in the public sector, while below 3 is considered efficient for the private sector [19].

To assess affordability, ten common diseases in Malaysia were selected. The affordability was computed using the daily wage of the lowest-paid unskilled government worker, which has been assessed as RM 16.03 per day (USD 4.18).

Medicine component costs were also recorded in the public, private, retail pharmacy and dispensing doctors' sectors in Kuala-Lumpur to assess pricing structure. For this, a separate form was developed and validated. Five medicines—atenolol 50 mg tablet (IB), atenolol 50 mg (generic), losartan 50 mg (IB), omeprazole 20 mg tablet (IB), and omeprazole 20 mg (generic)—were chosen on the basis of their widespread use and availability. The WHO–HAI methodology was followed to collect data on the different stages in the distribution chain [20]. Stage 0 of the component cost is manufacturer's selling price (MSP). Stage 1 includes stage 0 and insurance and freight. Stage 2 includes customs charges, port charges, and quarantine charges after the arrival of medicines in the country. It also includes finance, banking fees, and transport charges. Letter of credit charges are included in the finance and banking fees. Stage 3 includes distributors' and/or wholesalers' charges. Stage 4 is retailers' and dispensing doctors' markups. Stage 5 is composed of the value added tax and goods and services tax. As there are no value added tax, goods and services tax, or dispensing fees in Malaysia, data for stage 5 could not be collected. Data collection started with the patient/retail price; all the fees collected and the costs were deducted until a cost approximating the MSP was arrived at. All data on price components were collected in the Kuala Lumpur area of Malaysia. A description of the component cost stage by stage is given in Table S2.

## Results

In this study, 20 public hospitals, 32 private sector pharmacies, and 20 dispensing doctors' clinics were selected for data collection.

### Medicine Prices

In procurement for the public sector, the MPRs of 14 IBs were 2.41 times the IRPs, while for 26 MSG and LPG products, median MPRs were 1.56 and 1.09 times the IRP, respectively. The MPRs of four IBs—fluoxetine, loratadine, amlodipine, and simvastatin—were more than ten times the IRP. In private sector retail pharmacies (PSRPs), the median MPR for 32 IBs was 16.35 times the IRP, while the generics had median MPRs of 6.89 for 31 MSGs and 6.57 for 36 LPGs. The MPRs for four IBs in the private sector were over 50 (fluconazole, ciprofloxacin, furosemide, fluoxetine) with generic MPRs ranging from 10.9 to 39.3 for these four medicines. In the dispensing doctor sector (DDS) the median for the 17 IBs was 15.40 times the IRPs, while both the MSGs and LPGs were 7.76 times higher than the international reference prices. Some of the generics, such as metformin, hydrochlorothiazide, and isosorbide dinitrate were twice the price in dispensing doctors' clinics that they were in PSRPs. Table 2 summarizes the median MPRs of all the sectors studied.

**Table 2:**
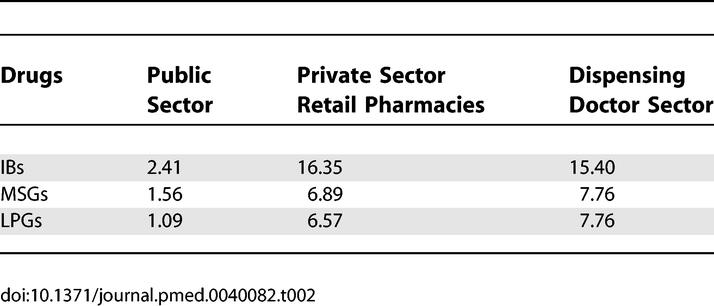
Median MPRs of IBs, MSGs, and LPGs in Public Procurement Sector, Private Sector Retail Pharmacies, and Dispensing Doctors' Sector

**Figure 1:**
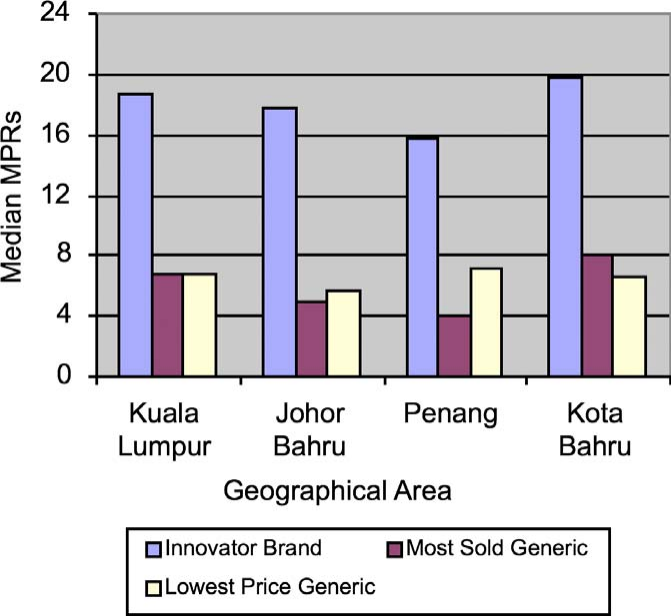
Regional Variation in Median MPRs in Private Retail Sector Pharmacies

Large variations in MPRs over the four geographical areas in private sector retail pharmacies were noted. The highest number of pharmacies were located in Federal Territory (Kuala Lumpur), followed by Johore Bahru, Penang, and Kota Bharu. Of all the drugs, the highest median MPR for IBs (19.83) was found in Kota Bharu followed by Kuala Lumpur (18.76), Johore Bahru (17.76), and Penang (15.82) (Figure 1).

Price comparisons were made only when drugs were found to be commonly available in all four regions. Thus, while 144 drugs were available overall, only 77 were common in every region. Kruskal-Wallis test indicated a nonsignificant (*p* = 0.748) difference in the prices of above 77 drugs (Table 3).

**Table 3:**
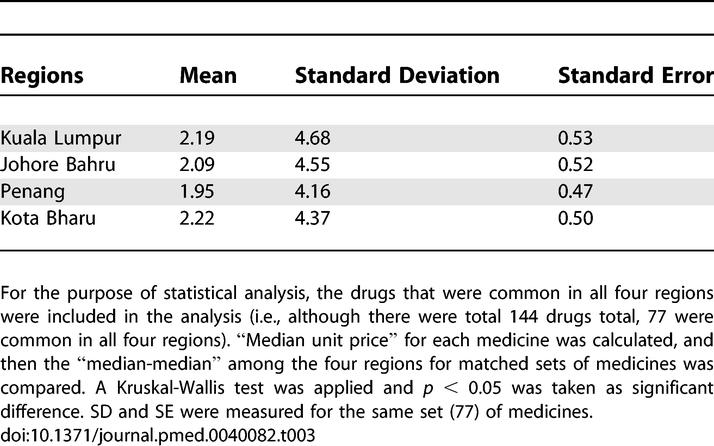
Mean Prices (RM) and Variances of the 77 Drugs Common in All Four Regions

The price variation in pharmacies appeared to have little linkage with the density of pharmacies, with the exception of Kota Bharu, where the number of pharmacies is the lowest but the drug costs are highest. Another reason for the highest prices could be distance: this state is far from Kuala Lumpur, the main distribution hub for pharmaceuticals. However, for the other states, the reasons for price differentials could not be determined with certainty; the only possible reason could be a “free price-setting” environment.

The MPR of IB amlodipine showed the highest variation, followed by that of IB glibenclamide and MSG ciprofloxacin. The MPR of metformin varied considerably; it was 3.9 times the IRP in Kuala Lumpur, 4.2 times in Johore Bahru, 3.4 times in Penang and 4.9 times (the highest) in Kota Bharu.

### Availability

In the public sector, median availability was very low, and only 25% of the generic drugs were available. In the private pharmacies, the median availability of all surveyed medicines was 43% for LPG, 18% for MSGs, and 39% for IBs. In dispensing doctors' clinics, the availability was 45% for LPGs, 15% for MSGs, and 10% for IBs.

The availability was also found low on the drugs, which are listed in the National Essential Drug List and the Drug Formulary of Malaysia [21,22]. For 41 medicines that were found in both sources, a combined analysis show that in the public sector, median availability was 40% for LPGs, 0% for MSGs, and 5% for IBs. In private sector retail pharmacies, median availability was 43.8% for lowest price generics, 18.8% for most sold generics and 40.6% for IBs. In the DDS, median availability was 45% for LPGs, 10% for MSGs, and 10% for IBs.

Low availability of antiviral drugs such as indinavir, nevirapine, and zidovudine was found in public hospitals, private pharmacies, and dispensing doctors' clinics. No private retail pharmacy was found to carry any version of diazepam 5 mg tablets or fluphenazine 25 mg/ml injection. Availability data can be found in Table 4.

**Table 4:**
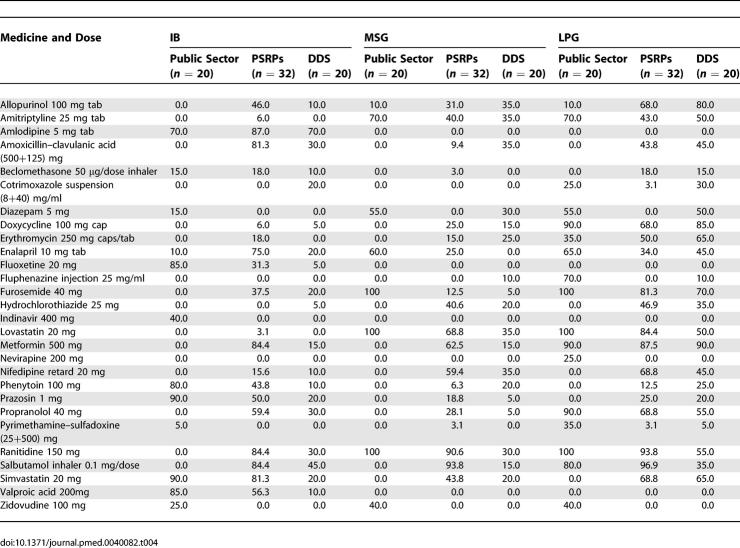
Median Percentage Availability of Selected Medicines in the Public Sector, Private Retail Pharmacy Sector, and Dispensing Doctors' Sector

### Affordability

As medicines are provided free in the public sector, affordability has been assessed only for the private sector. A one-month treatment of IB ranitidine (150 mg twice daily) for peptic ulcer required 7.5 days' wages when purchased from private pharmacies and 8 days' wages from dispensing doctors' clinics. The cost of generic versions of ranitidine was 3 days' wages in the pharmacies and 3.7 days' wages from the dispensing doctors' clinics. IB omeprazole (20 mg daily) cost 14–15 days' wages for a one-month treatment, while its LPG cost about 3–4 days' wages in both sectors. The cost of a one-month treatment with IB amlodipine (5 mg daily) required about 4.9 days' wages. To buy simvastatin (20 mg daily), the patient had to pay 7.5 days' wages in private pharmacies and 6 days' in dispensing doctors' clinics. Purchasing generic simvastatin cost about 2.3 days' wages in both sectors.

**Figure 2:**
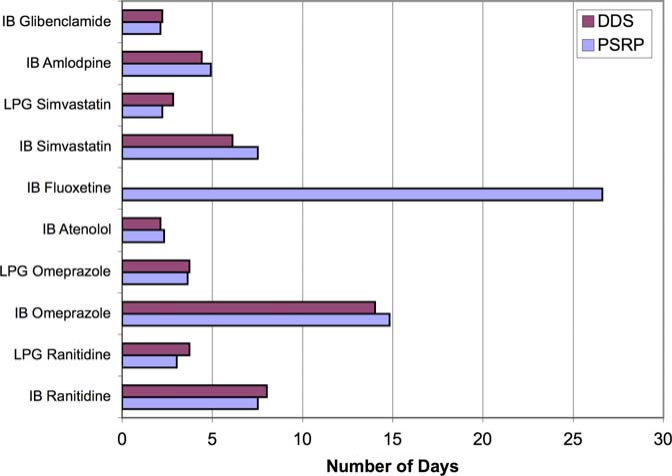
Affordability of Standard Treatments in the Dispensing Doctors' Sector and Private Sector Retail Pharmacies

Fluoxetine (20 mg daily) cost about 26 days' wages for one month of treatment when purchased at private pharmacies. IB acyclovir for acute viral infections (200 mg fives time daily for 5 days) cost patients 8 days' wages, while generic cost 3 days' wages. Patients have to pay 2 days' salary to buy the IB glibenclamide, while for generic products they have to spend approximately half a day's salary. Figure 2 shows the affordability data in the DDS and PSRP for selected drugs.

### Price Components

#### Procurement for public sector.

Generic atenolol in stage 1 (MSP + insurance and freight) was found to be 68% of the total cost while it was 79% for IB atenolol. The total markups (in all stages) for atenolol were 47%and 27%, respectively, for its generic and IB. For generic omeprazole stage 1 contributed 81% while the total markups were 26%. For IB omeprazole, stage 1 was 84% and the markups were 18%. For losartan the stage 1 component price was 83%, while total markups were 20% (Table 5).

**Table 5:**
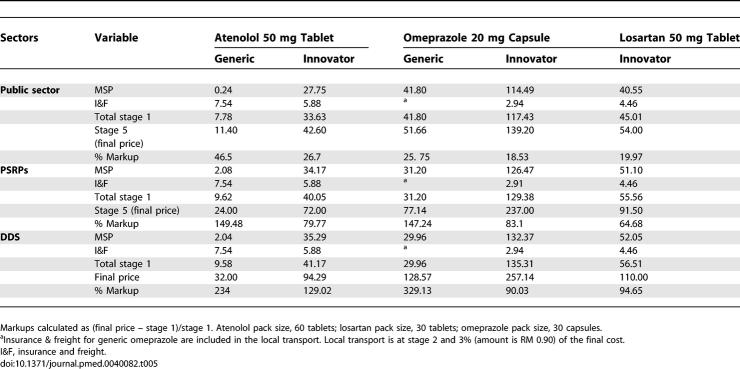
Summary and Markups for Different Drugs in Public Sector, PSRPs, and DDS

#### Private sector retail pharmacies.

The markups of generic atenolol were 150% with stage 1 being only 40% of the final price. For IB atenolol, the markups were 80%, with the base price 56% of the final cost. For generic and IB omeprazole, the markups were 147% and 83% and stage 1 was 41% and 56%, respectively. For losartan, stage 1 was 61% and markups were 65% (Table 5).

#### Dispensing doctors' sector.

In the DDS, stage 1 was 30% for generic atenolol and 43% for IB atenolol. The markups of atenolol were 234% and 129% for generic and IB, respectively. For IB omeprazole, stage 1 was 53% of the final cost and the markups were 90%. For generic omeprazole, stage 1 was 24% and total markups were 329% (Table 5). As indicated in Table 6, for generic omeprazole, the manufacturing price for a pack of 30 capsules was 29.96 RM, and after the addition of a 3% local transport cost, it became 30.86 RM; it jumped to 128.57 RM with a retailer mark up of 316%. For IB losartan, stage 1 was 51% and markups were 94% (Table 5).

**Table 6:**
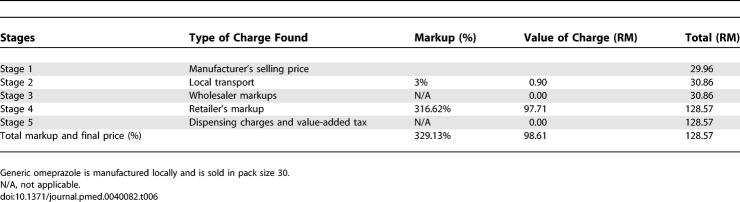
Dispensing Doctors' Sector Component Costs for Generic Omeprazole 20 mg Capsule

## Discussion

In Malaysia, public sector procurement prices were high for IBs, and both IBs and generics were very expensive in DDSs and PSRPs when compared with the IRPs. Prices varied across sectors in private sector retail pharmacies.

Malaysian medicine prices were very high in terms of international pricing (the IRP). The Malaysian dataset was also compared with India and Sri Lanka as these countries have shown efficient procurement and pricing. In Indian state of West Bengal, in public procurement sector, a median MPR of 0.75 has been reported for generics while in private retail pharmacies a median MPR of 2.86 and 2.17 has been observed for IBs and LPGs, respectively [23]. In the Indian state of Rajasthan, for generic medicines a median MPR of 0.96 was noted in the public procurement sector, while in PSRPs median MPRs of 2.81 and 1.83 were recorded for IBs and LPGs, respectively [24]. A similar situation was found in Sri Lanka, where a median MPR of 2.67 was noted for IBs and 0.82 for LPGs in PSRPs [25].

Low availability of medicines on the National Essential Drug List and the Drug Formulary [21,22] were found in all sectors, particularly in the public sector. Poor availability of generics was also seen in the public sector. The low availability of medicines at public hospitals could have direct implications on access, as patients are then forced to buy these medicines from private pharmacies or dispensing doctor clinics. Private pharmacies carried fewer generic drugs than did the dispensing doctors, and thus they may dispense more IBs. Due to nonavailability of many drugs, many patients now dig deep into their pockets to pay for medicines [26]. Better availability in the public sector would put pressure on private sector to lower generic prices. Dispensing doctors tend to prescribe generics, but they charge excessive markups for generics compared to IBs.

Psychotropics such as diazepam and fluphenazine were not found in any of the retail pharmacies, probably due to the stricter regulatory requirements of their drug licences. However, these medicines were available at dispensing doctors' clinics and public facilities. Generic versions of fluoxetine and amlodipine were not available in the market, as these drugs are still under patent in Malaysia.

Affordability data indicated that a large part of the population would not be able to pay for their medicines. Some diseases such as cardiovascular disorders are on the rise in Malaysia [27]. Some common drugs to treat these conditions are amlodipine and simvastatin, and patients have to pay between 5–7 days' wages to buy one month's treatment with these drugs. Mental illness has become the fourth leading cause of morbidity in the country, but medicines such as fluoxetine cost about 26 days' wages for one month's treatment, and there is no generic version available for this medicine. To treat common diseases such as diabetes and viral illnesses, patients have to pay 2 days' and 8 days' wages to buy the innovator versions of glibenclamide and aciclovir, respectively.

Affordability of generics also seems to be an issue in the DDS and PSRP. To be able to purchase generic aciclovir and simvastatin, patients have to work for 2 to 3 days. The unaffordability of these medicines could pose problems for public health.

### Price Components

Add-on costs had a substantial impact on medicine prices in all sectors in Malaysia. In retail pharmacies, actual markups of 100%–140% were found for generics, and 25%–38% for innovators.

The Malaysian markups were compared with those in other countries where the WHO–HAI surveys have been published, and found to be higher in Malaysia. For example, in Sri Lanka's private for-profit pharmacy sector, the wholesale markup is 8% and retailer's markup is 16%. In Kenya, the private retail sector (for imported medicine) has a wholesale markup of 15%–30% and a maximum retailer markup of 20%–33%. In Peru, for imported generic medicine the distributor's markup is 36% while the retailer's markup is 33%. Armenia's private sector (for imported medicines) wholesaler/distributor markup ranges from 18% to 25% while the retailer's markup is 15%–25%. In Brazil, in the private retail sector the wholesaler's and retailer's markup is around 27%. In the Philippines' private retail sector, the distributor and retailer markup for IBs is 30%, and for locally manufactured generics the retailer's markup is about 100% [28]. In the Indian state of Maharashtra, a profit margin of 20% was found for generic atenolol [29] compared to 100% in Malaysia.

Add-on costs for both IBs and generic equivalents were higher in the DDS than the other two sectors. For IBs dispensing doctors' markups ranged from 50% to 76%, while for generics they were up to 316% (generic omeprazole). In the Malaysian health care system, patients rely heavily on physicians' advice and recommendations. Profit margins and markups in this sector are particularly high for generics as compared to IBs.

Generally, high markups along the supply chain drive up prices and make medicines less affordable; therefore prices of medicines can be lowered substantially by reducing the markups.

Dispensing doctors are taking advantage of lower priced generics and are marking them up to make a larger profit. Some generics, such as atenolol, were still less expensive than their innovator and even after a significant markup leave them considerably more affordable—than the IB. However, for generic omeprazole markups were found to be excessive. This trend of increased profits could lead to irrational drug use, as has been observed in Zimbabwe, where a desire to increase income was associated with less clinically and economically appropriate prescribing [30].

Components analysis indicated high MPRs on the costliest IBs, principally reflecting high manufacturers' prices. The IB prices remain unaffected even in the presence of generic competition because they are being sold with progressive profits to the manufacturers, distributors, and retailers. Generics are more affordable than IBs, and they could be even more so if markups were restricted and there were incentives to encourage increased use of generics.

### Economic Analysis

Medicine price determination is a complex process, and most national markets are highly segmented with different procedures applying in different sectors and to different medicines. “Free markets” do not apply with medicines under patent, as these by definition confer a degree of market exclusivity to the manufacturer. Manufacturers frequently set different prices in different markets even within the same country. In Malaysia, for IB medicines, generally the conditions for a properly functioning market are not met, as there is a supplier monopoly for these particular medicines. The actual situation in Malaysia differs from sector to sector as well as by type of medicine. Branded generics, the most numerous generics, are more akin to other branded commodities, in that they compete with each other and are therefore subject to normal supply and demand pressures in determining their prices.

The higher markups on generics observed in the components analysis suggest that the prices of innovator drugs are used as a limit to generic pricing. In such a situation, price-controlled generics may be a sensible way out of the problem. Malaysia has no history of price control on retail medicines, and indeed the current commercial ethos of the country is toward market mechanisms. Recent research suggests that increasing the available choices of generics on the market does not, on its own, bring down prices [17]. Nevertheless, printed maximum retail prices on packs of selected generic medicines (as in India), under the oversight of the Pharmaceutical Services Division of the Ministry of Health, could be used as a mechanism to ensure that retail prices do not diverge excessively from IRPs. In the process, this would put downward pressure on other generic prices and on the prices of IBs (by raising the price differential or “brand premium”).

Overall high prices in Malaysia, compared to the reference prices, might be due to a relatively unregulated system [31,32]. The Malaysian government is encouraging the pharmaceutical industry to grow, but the degree to which the market should be allowed to determine drug prices is a problematic issue [33]. Nevertheless, the growing burden of medicine spending on poorer households cannot be ignored, nor can costly medicines be dismissed as a problem of inappropriate prescribing when retail generics from private sector prescribers cost six to eight times international bulk purchase prices. Any scheme of differential pricing should recognize this, and purchasing bodies in Malaysia might make effective use of such evidence in their discussion on pricing with suppliers.

### Pricing Models in Other Countries

Therefore, government involvement in pharmaceutical pricing practices is necessary because leaving the financing and supply of drugs entirely to the market economy may also fail to achieve public health objectives [2]. Pricing regulations can be found in most of the European and Middle Eastern countries, Australia, New Zealand, the Far East, and Canada [34]. Some countries, such as France and Italy, regulate drug prices directly through price control; others, such as Australia, use pharmacoeconomic analyses and reference pricing to determine the prices of drugs subsidized by the government [35,36]. Australia and the province of Ontario in Canada were also the first to include cost effectiveness data in decisions about reimbursement [37].

Germany and Japan control prices indirectly through limits on reimbursement under social insurance schemes [35]. In the UK, the National Pharmaceutical Pricing Authority monitors prices through the Pharmaceutical Price Regulation Scheme, which controls the prices of branded prescription medicines to the National Health Service by regulating the profits that companies can make on sales [38]. In The Netherlands, the government introduced reference-pricing system in 1991, and wholesalers were forced to lower their prices by an average of 20% in 1996 [39]. In Finland, the application of marketing authorization must contain a detailed assessment of cost of the drug therapy and the prices of medicines [40]. Pricing regulations also seem vital, as in many Latin American countries “free schemes of drug prices” have not proved effective as a cost containment mechanism. In Guatemala and Peru, prices have increased over and above the exchange rate or the consumer price index, and in Bolivia the practice of the free market concept has not yielded any benefits [41].

In India, essential drugs cannot cost more than twice the cost of production, and the maximum retail price and local taxes must be included in a drug's final printed price [42]. This model follows the “cost-plus pricing” where the prices are negotiated between the manufacturer and the national authority, based on raw material costs, production costs, marketing costs, and a reasonable allowance for profit [2]. The Canadian Patented Medicines Prices Review Board sets maximum introductory prices for newly patented medications, and forecasts and regulates the prices of medicines by calculating a consumer price index adjustment factor [43,44].

However, Malaysia, a middle-income developing country, so far practices none of the pricing methods mentioned above. Price controls can also be implemented in a variety of ways, some of which cause less distortion in the medicines market than others. Perhaps the most efficient and effective is to use, in purchasing, price benchmarks in countries at comparable economic levels, to ensure that a country is not becoming a “high price island.” Reference pricing sets or limits the price of an individual drug by comparison with the price of other drugs in other countries [2].

Access to affordable and lower priced medicines are the aims of Draft National Medicine Policy of Malaysia, but according to this study's findings, these aims are not being achieved [45]. A medicine pricing policy and a price monitoring system is required in Malaysia. Prices may also be controlled by fixing margins of retailers and wholesalers, and enforced by marking maximum prices on packs. On the procurement side, a gallery of price-reducing strategies, from bulk purchasing, evidence-based negotiation based on cost-effectiveness, parallel trading, or differential pricing may be employed to put downward price pressure on manufacturers. There must be incentives to promote the use of generics, and some form of mandatory generic substitution is also required.

### Limitations of the Study

Some of the drugs studied, such as fluconazole, amoxicillin–clavulanic acid, captopril, ciprofloxacin, nifedipine retard, and zidovudine were found in different strengths from what were specified in the medicine price data collection form. As a result, they were not recorded. Therefore, nonavailability and lower availability of these drugs may not be meaningful, because they may be available but in a different strength.

Also, in the public sector, availability of drugs was low overall, and moreover the drugs, which were available only in four or more facilities, were included in the analysis. Therefore, low availability of drugs in the public sector also makes the median MPR less robust.

### Conclusion and Recommendations

Expecting an unregulated and free market system to ensure access to essential medicines in Malaysia seems unrealistic. Prices were found to be generally high in PSRPs and dispensing doctor clinics, for IBs as well as generics. In the public sector availability was low even for the medicines on the National Essential Drugs List. Low affordability was observed even for the drugs use for some common ailments, such as hypertension, asthma, respiratory disorders. Generally high markups and profit margins were noted in dispensing doctors and private retail sectors for generics and IBs. The MSP was high for innovator brands as compared to their generics in all sectors.

A pricing policy is needed, and it should be incorporated into the national drug policy. This policy should aim to improve the availability of affordable generics. A package of measures, some of them incremental or experimental, suggests itself on the basis of the above findings. Such measures would include ensuring better availability in the public sector, either by better targeting of existing public spending for medicines on priority medicines, or by increasing the public budget for essential medicines; this in turn would put downward pressure on private sector generic prices. The package would also review purchasing practices to ensure greater efficiency. And although dispensing doctors may be useful as prescribers of generic drugs, they have incentives to overcharge because of the high cost of IB medicines. Reducing the brand premium by better purchasing strategies and by experimenting with the control of selected generic prices could be other options.

A price monitoring system is also needed in Malaysia. The availability of HIV drugs must also be ensured. A generic policy should also be devised that should include campaigns to promote generics, increase consumer awareness, and introduce incentives for pharmacists and doctors to prescribe and dispense generics.

## Supporting Information

Table S1 Drugs Surveyed in This Study (A) List of the core drugs surveyed.
(B) List of supplementary drugs surveyed.
(49 KB DOC)

Table S2 General Overview of Component Costs by Stages (53 KB DOC)

## Abbreviations

DDS: dispensing doctors' sector
HAI: Health Action International
IB: innovator brand
IRP: international reference price
LPG: lowest-price generic
MPR: median price ratio
MSG: most-sold generic
MSP: manufacturer's selling price
PSRP: private sector retail pharmacy
RM: Malaysian ringgit
WHO: World Health Organization
