# Supplementary material for: Evaluating Drug Prices, Availability, Affordability, and Price Components: Implications for Access to Drugs in Malaysia
Source: PLoS Med. 2007 Mar 27;4(3):e82. doi: 10.1371/journal.pmed.0040082 (PMC1831730; doi:10.1371/journal.pmed.0040082)
Supplement: Table S1 — (A) List of the core drugs surveyed. (B) List of supplementary drugs surveyed. (49 KB DOC) [file pmed.0040082.st001.doc]

| **No.** | **Drugs** | **No.** | **Drugs** |
| --- | --- | --- | --- |
| 1 | Acyclovir 200mg tab | 15 | Glibenclamide 5mg tab |
| 2 | Amitriptyline 25mg tab | 16 | Hydrochlorothiazide 25mg tab |
| 3 | Amoxicillin 250mg caps/tab | 17 | Indinavir 400mg caps |
| 4 | Atenolol 50mg tab | 18 | Losartan 50mg tab |
| 5 | Beclometasone 50mcg/dose inhaler | 19 | Lovastatin 20mg tab |
| 6 | Captopril 25mg tab | 20 | Metformin 500mg tab |
| 7 | Carbamazepine 200mg tab | 21 | Nevirapine 200mg tab |
| 8 | Ceftriaxone 1gm powder for injection | 22 | Nifedipine Retard 20mg tab |
| 9 | Ciprofloxacin 500mg tab | 23 | Omeprazole 20mg caps |
| 10 | Co-trimoxazole (8+40) mg/mL paed suspension | 24 | Phenytoin 100mg caps/tab |
| 11 | Diazepam 5mg tab | 25 | Pyrimethamine + sulfadoxine (25+500) mg tab |
| 12 | Diclofenac 25mg tab | 26 | Ranitidine 150mg tab |
| 13 | Fluoxetine 20mg caps/tab | 27 | Salbutamol 0.1 mg/dose inhaler |
| 14 | Fluphenazine decanoate 25mg/mL injection | 28 | Zidovudine 100mg caps |

## Table S1 (a) List of the Core Drugs surveyed

| **No.** | **Drugs** | **No.** | **Drugs** |
| --- | --- | --- | --- |
| 1 | Allopurinol 100mg tab | 11 | Isosorbide dinitrate 10mg tab |
| 2 | Amlodipine 5mg tab | 12 | Itraconazole 100mg caps/tab |
| 3 | Amoxicillin+Clavulanic acid (500+125) mg tablet | 13 | Loratadine 10mg tab |
| 4 | Dimenhydrinate 50mg tab | 14 | Metoclopramide HCl 10mg tab |
| 5 | Doxycycline 100 mg caps/tab | 15 | Prazosin 1mg tab |
| 6 | Enalapril 10mg tab | 16 | Prednisolone 5mg tab |
| 7 | Erythromycin 250mg caps/tab | 17 | Propranolol 40mg tab |
| 8 | Fluconazole 150mg tab | 18 | Simvastatin 20mg tab |
| 9 | Furosemide 40mg tab | 19 | Spironolactone 25mg tab |
| 10 | Ibuprofen 200mg tab | 20 | Valproic Acid 200mg tab |

# Table S1 (b) List of Supplementary Drugs surveyed
