# Supplementary material for: Evaluating Drug Prices, Availability, Affordability, and Price Components: Implications for Access to Drugs in Malaysia
Source: PLoS Med. 2007 Mar 27;4(3):e82. doi: 10.1371/journal.pmed.0040082 (PMC1831730; doi:10.1371/journal.pmed.0040082)
Supplement: Table S2 — (53 KB DOC) [file pmed.0040082.st002.doc]

|  | **International Import** | |  | **Local Production** | |
| --- | --- | --- | --- | --- | --- |
| Stage 1 | Manufacturer’s Selling Price (MSP) | Value |  | Manufacturer’s Selling Price (MSP) | Value |
|  | Insurance & Freight (CIF) | Value |  | Transport | n/f |
|  |  |  |  |  |  |
| Stage 2 | Finance/Banking Fees | Value |  | Finance/Banking Fees | n/f |
|  | Incidental/Other Banking Fees | Value |  | Incidental/Other Banking Fees | n/f |
|  | International Inspection Fee | n/a |  | Local Transport | Value |
|  | Pharmacy Board Fee | n/a |  | Pharmacy Board Fee | n/a |
|  | Port Charges & Clearance Fees | Value |  | National Taxes | n/a |
|  | Import Tariff | n/a |  |  | |
|  | Importer’s Mark-Up | Value |  |  |  |
|  |  |  |  |  |  |
| Stage 3 | Distributor’s/Wholesaler’s Mark-Up | Value |  | Distributor’s/Wholesaler’s Mark-Up | Value |
|  | Regional Taxes | n/a |  | Regional Taxes | n/a |
|  | Transport | n/f |  | Transport | n/f |
|  |  |  |  |  |  |
| Stage 4 | Retailer’s/Dispensing Doctors’ Mark-Up | Value |  | Retailer’s/Dispensing Doctors’ Mark-Up | Value |
|  | Local Taxes | n/a |  | Local Taxes | n/a |
|  |  |  |  |  |  |
| Stage 5 | VAT/GST | n/a |  | VAT/GST | n/a |
|  | Dispensing Fees | n/a |  | Dispensing Fees | n/a |
| *n/a: Not Applicable; Price Component Does Not Exist*  *n/f: Not Found; Price Component Exist But Data Not Found*  *Value: Price Component Exist and Value Found* | | | | | |

**Table S2: General overview of component costs by stages**
